# The Clinically Actionable Molecular Profile of Early versus Late-Stage Non-Small Cell Lung Cancer, an Individual Age and Sex Propensity-Matched Pair Analysis

**DOI:** 10.3390/curroncol29040215

**Published:** 2022-04-11

**Authors:** Anna L. McGuire, Melissa K. McConechy, Barb L. Melosky, John C. English, James J. Choi, Defen Peng, John Yee, Benjamin L. S. Furman, Rosalia Aguirre Hernandez, Pedro Feijao, David Mulder, Curtis Hughesman, Stephen Yip

**Affiliations:** 1 Vancouver Coastal Health Research Institute, 7113-2775 Laurel Street, Vancouver, BC V5Z 1M9, Canada; john.english@vch.ca (J.C.E.); john.yee@vch.ca (J.Y.); 2 Vancouver General Hospital, 899 West 12th Avenue, Vancouver, BC V5Z 1M9, Canada; james.choi@vch.ca (J.J.C.); syip-02@bccancer.bc.ca (S.Y.); 3 Canexia Health Inc., 1-3661 West 4th Avenue, Vancouver, BC V6R 1P2, Canada; mmcconechy@canexiahealth.com (M.K.M.); bfurman@canexiahealth.com (B.L.S.F.); raguirre@canexiahealth.com (R.A.H.); pfeijao@canexiahealth.com (P.F.); dmulder@canexiahealth.com (D.M.); 4 BC Department of Medical Oncology, BC Cancer—Vancouver Centre, 600 West 10th Avenue, Vancouver, BC V5Z 4E6, Canada; bmelosky@bccancer.bc.ca; 5 Centre for Health Evaluation and Outcome Sciences (CHEOS), 588-1081 Burrard Street, Vancouver, BC V6Z 1Y6, Canada; defen.peng@gmail.com; 6 Cancer Genetics & Genomic Laboratory, BC Cancer—Vancouver Centre, 600 West 10th Avenue, Vancouver, BC V5Z 4E6, Canada; curtis.hughesman@bccancer.bc.ca

**Keywords:** lung cancer, next-generation sequencing, biomarker, targeted therapy

## Abstract

Background: Despite meticulous surgery for non-small cell lung cancer (NSCLC), relapse is as high as 70% at 5 years. Many institutions do not conduct reflexive molecular testing on early stage specimens, although targeted gene therapy may extend life by years in the event of recurrence. This ultimately delays definitive treatment with additional biopsy risking suboptimal tissue acquisition and quality for molecular testing. Objective: To compare molecular profiles of genetic alterations in early and late NSCLC to provide evidence that reflexive molecular testing provides clinically valuable information. Methods: A single-center propensity matched retrospective analysis was conducted using prospectively collected data. Adults with early and late-stage NSCLC had tissue subject to targeted panel-based NGS. Frequencies of putative drivers were compared, with 1:3 matching on the propensity score; *p* < 0.05 deemed statistically significant. Results: In total, 635 NSCLC patients underwent NGS (59 early, 576 late); 276 (43.5%) females; age 70.9 (±10.2) years; never smokers 140 (22.0%); 527 (83.0%) adenocarcinomas. Unadjusted frequencies of EGFR mutations were higher in the early cohort (30% vs. 18%). Following adjustment for sex and smoking status, similar frequencies for both early and late NSCLC were observed for variants in EGFR, KRAS, ALK, MET, and ROS1. Conclusion: The frequency of clinically actionable variants in early and late-stage NSCLC was found to be similar, providing evidence that molecular profiling should be performed on surgical specimens. This pre-determined profile is essential to avoid treatment delay for patients who will derive clinical benefit from targeted systemic therapy, in the high likelihood of subsequent relapse.

## 1. Introduction

### 1.1. Background

Each year lung cancer kills more people than colon, breast, and prostate cancers combined [1]. In fact, 26% of all cancer-related deaths are attributable to lung cancer [1]. The World Health Organization (WHO) classifies lung cancer into two broad categories on the basis of tumor biology, treatment, and prognosis: non-small cell lung cancer (NSCLC) and small cell lung cancer (SCLC) [2,3]. The only patients with a prospect of being cured are those with early (stage I/II/IIIA) NSCLC who are amenable to surgical resection [4]. Molecular testing with next-generation sequencing (NGS) of resected early-stage NSCLC for clinically actionable genetic alterations is not routinely conducted in many institutions following standard of care curative intent pulmonary resection. Adjuvant targeted systemic therapy is currently only recently reserved for select cases of EGFR (epidermal growth factor receptor) sensitizing mutations based on demonstrated benefit in 36-month relapse free survival [5]. Further targeted systemic therapy is limited to non-resectable advanced stage patients with tumor genetic alterations such as ALK (anaplastic lymphoma kinase), MET (Met proto-oncogene), RET Ret proto-oncogene), and ROS1 (c-ROS proto-oncogene 1) [6,7,8,9,10,11,12,13,14,15]. Novel targeted therapeutics are also under development for KRAS G12C (Kirsten rat sarcoma viral oncogene homolog) [11,16,17]. Despite undergoing curative intent surgery for early-stage disease, up to 70% of patients with early-stage NSCLC at presentation still suffer recurrent metastatic disease [1]. NSCLC patients with recurrent metastatic disease are subsequently eligible for targeted systemic therapy if the tumor expresses clinically actionable genetic alterations such as EGFR, ALK, MET, RET, and ROS1. They are furthermore potentially eligible for participation in clinical trials involving KRAS G12C targeted therapeutics. Prior studies of NGS in early-stage lung cancer have been conducted demonstrating that putative driver mutations present at the time of surgical resection portend prognosis [18]. Additionally, although an element of subclonal heterogeneity may be present at the time of recurrent lung cancer following initial curative intent surgery, the dominant clinically relevant clonal “oncogenic driver” identified at the time of surgery was also present at the time of recurrence conferring sensitivity to targeted therapy [19]. At the time of recurrent disease, technical challenges often exist with respect to the ability to biopsy and acquire sufficient quantities of malignant tissue to undergo robust molecular profiling with NGS [15]. We hypothesized that both early and late-stage NSCLC display similar odds of driver mutation and fusion molecular alteration profiles in our population regardless of disease stage at presentation. This study would provide clinical rationale for routine targeted panel next-generation sequencing testing of all resected early-stage NSCLC formalin fixed paraffin embedded (FFPE) surgical specimens, providing crucial knowledge that could alter postoperative treatment options for this life-threatening disease.

### 1.2. Objective

The objective of the current study is to compare the molecular profiles of clinically actionable genetic alterations in early and late non-small cell lung cancer in our cohort.

## 2. Methods

### 2.1. Study Design and Setting

A propensity matched cohort study was conducted at the BC Cancer (Vancouver Cancer Centre or VCC) and the Vancouver General Hospital (VGH) Division of Thoracic Surgery. Tumor molecular data derived during the time period August 2019 to August 2021. This study, #H18-03295-A009, was approved by the BC Cancer Institutional research ethics board.

### 2.2. Participants and Data Sources

Prospectively collected molecular, demographic, and smoking status variables for consecutive cases of early and late-stage NSCLC were retrospectively analyzed. The early-stage NSCLC dataset was derived from that collected as part of a separate pilot study database assessing the use of targeted cancer gene panels in early-stage NSCLC patients. Early-stage NSCLC patients were eligible for targeted molecular testing as part of this study following surgical resection for early stage I/II NSCLC (American Joint Commission on Cancer [AJCC] Staging 8th edition) [4] if their tumor was ≥10 mm in maximal diameter and solid in morphology on preoperative CT chest. The early-stage NSCLC patient group additionally underwent standard of case physiologic assessment including detailed pulmonary function testing and clinical staging with fluorodeoxyglucose positron emission tomography (FDG-PET) scan [15]. A selective approach to preoperative invasive mediastinal staging was undertaken. The late-stage NSCLC variables were derived from the Cancer Genetics & Genomics Laboratory (CGL) testing database of patients who presented to BC Cancer for management on unresectable late-stage NSCLC.

### 2.3. Primary Outcomes

The primary outcomes of interest were frequency of clinically actionable lung tumor genetic alterations in the early and late-stage cohorts. Potential confounding variables of interest identified from the literature included sex, age, and tobacco smoking status (current, former, never) [15].

### 2.4. Targeted NGS Panels for NSCLC Genetic Alterations

For both the early and late-stage cohorts, the determination of clinically actionable molecular alteration status was conducted using formalin fixed paraffin embedded (FFPE) tumor tissue.

Early stage surgically resected NSCLC FFPE slides were assessed for tumor content by a thoracic pathologist and sent to the Canexia Health laboratory for DNA/RNA extractions and sequencing. DNA was extracted using the Qiagen Generead kit and UNG treatment using the manufacturer’s instructions. RNA was extracted using the Promega RNA FFPE kit using the Promega Maxwell RSC instrument. The Canexia Health Find ItTM assay, a FPPE solid tumor DNA-based assay, and the RNA-based Fusions assay was performed on the early stage surgically resected NSCLC FFPE tumor tissue specimens. The Find It assay is an amplicon-based targeted multiplex NGS test that is focused on clinically actionable hotspot gene content for detection of single nucleotide variants (SNVs) and insertions and deletions (indels). The fusions assay is an RNA-based gene partner agnostic multiplex NGS panel that can identify clinically relevant structural rearrangements or gene fusions. In brief, the Find It assay amplifies FFPE DNA in 2–3 separate primer pools using maximum 25 ng DNA in each pool. PCR template products were then pooled, purified, and amplified with Nextera XT Index kit V2 adapters or IDT for Illumina UDIs (unique dual indexes) for sequencing using the Illumina MiSeq v2 300 cycle kits. The in-house developed Canexia Health cloud-based bioinformatics pipeline uses BWA to align to the human reference genome GRCh37/hg19, undergoes multiple data filtering and QC, then utilizes artificial intelligence models trained to identify SNVs to variant allele frequencies (VAFs) of <1%. Post alignment, indels were analyzed using Strelka [20]. The fusions assay briefly uses reverse transcription for the conversion of FFPE derived RNA to cDNA. The cDNA was then subjected to amplification, ligation, then PCR for targeted amplification. The libraries were amplified using Illumina UDI adapters, purified and sequenced using the Illumina MiSeq v2 300 cycle kits. The in-house developed fusions analysis pipeline and algorithm identifies total unique fusion reads to identify high, medium, and low confidence fusion events. Immunohistochemistry was used to orthogonally validate the ALK gene fusion event.

The DNA-based hybrid-capture multiplex NGS assay (“oncopanel”) from the Cancer Genetics & Genomic Laboratory (CGL) at BC Cancer was utilized for the late-stage NSCLC cohort. Genomic DNA was extracted with an automated system (Promega Maxwell) followed by FFPE repair, ligation-based library construction, PCR amplification, hybridization capture, and sequencing on a HiSeq2500 platform. Single-strand consensus sequences are generated from UMI-indexed reads using fgbio and aligned to the GRCh37 human genome reference using BWA. Variant calling of DNA mutations and insertions/deletions (INDELs) was performed using samtools and VarScan2. Annotation and filtering of variants is performed with Agilent’s Alissa Interpret platform. For gene fusions in the late-stage cohort, immunohistochemistry was employed to determine aberrant protein expression of ALK, RET, and ROS1 status from matched FFPE slides.

### 2.5. Statistical Methods

Continuous variables were summarized by mean and standard deviation and analyzed using the Student’s *t*-test. Categorical variables were expressed as frequencies and percentages and compared by Chi-square or Fisher’s exact tests if appropriate. Single-factor and multi-factor analyses in relation to outcomes (e.g., EGFR) required the use of the logistic regression.

A propensity-matched comparison was conducted to control for potentially confounding variables. Age, sex, smoking status, and tumor characteristics were used in a logistic regression model to generate a propensity score for each patient with early stage of NSCLC or late stage of NSCLC. The matched cohort was derived using 1:3 matching with a maximum allowable absolute difference between the propensity scores of 0.20. The type of matching optimization was by closeness. The quality of the matching was assessed by using the standardized mean difference as well. A robust variance estimator was used to account for the clustering within matched sets when using a logistic regression model to compare the variables or to regress the outcomes on the stage of NSCLC.

The conventional level of statistical significance (*p* < 0.05) is used throughout the study as an indicator of a potential effect. All tests were two-sided. Additionally, all statistical analyses were performed with SAS software version 9.4 (SAS Institute, Cary, NC, USA).

## 3. Results

The final study cohort included 635 NSCLC patient samples (59 early stage and 576 late-stage) with targeted panel NGS-based molecular profiling for tumor mutations and fusions. The early-stage group included 22 (37.3%) females, 21 (35.6%) never smokers, and 49 (83.1%) adenocarcinoma histology, with a mean age of 68.0 (±10.3) years. The late-stage group was composed of 254 (44.1%) females, 119 (20.7%) never smokers, 478 (83.0%) adenocarcinomas, and a mean age of 71.2 (±10.2) years. A total of 17 late-stage patients missing smoking status data were excluded from the final analysis. Of the major NSCLC histologic WHO sub-groups, we reported 527 (83.0%) adenocarcinomas, 19 (3.0%) squamous cell carcinoma, and 89 (14.0%) other pulmonary carcinomas such as large cell and adenosquamous carcinoma. Baseline characteristics by stage of NSCLC among all patients are depicted in Table 1.

Using the propensity-matched comparison method, we identified a total of 53 out of the 59 patients from the early-stage group that matched with 159 of the 576 patients from the late-stage group. A total of 212 patients in the matched cohort were obtained. Baseline characteristics by stage of NSCLC among matched patients are depicted in Table 2. Molecular alteration outcomes by NSCLC stage group are presented in Table 3 for all patients and Table 4 for the propensity-matched patients.

**Figure 1:**
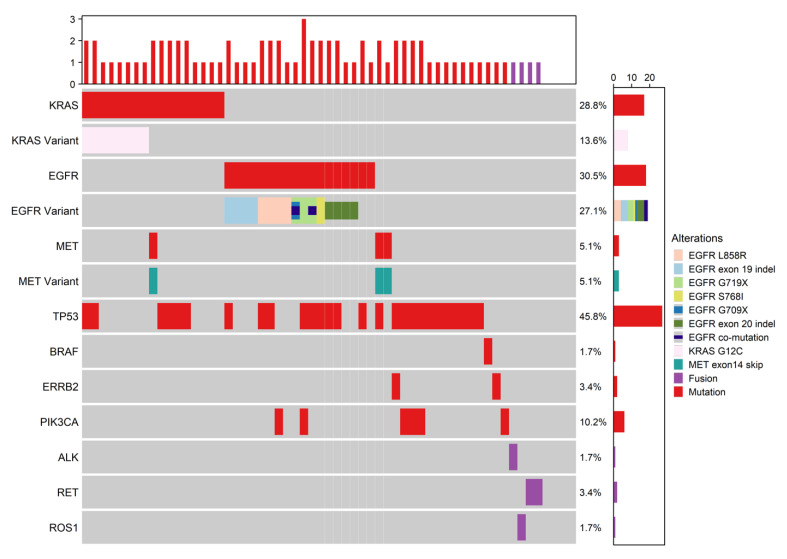
Oncoprint of the early stage NSCLC cohort genomic alterations. This oncoprint depicts genetic alterations identified by the Canexia Health Find It^TM^ assay, a FPPE solid tumor DNA-based assay, and the RNA-based Fusions assay. On the right, the alteration frequency per gene is reported, with the corresponding gene labelled on the left side. The barplots beside the gene labels report the alteration counts. The topmost bar represents the mutation and fusion count per sample.

**Figure 2:**
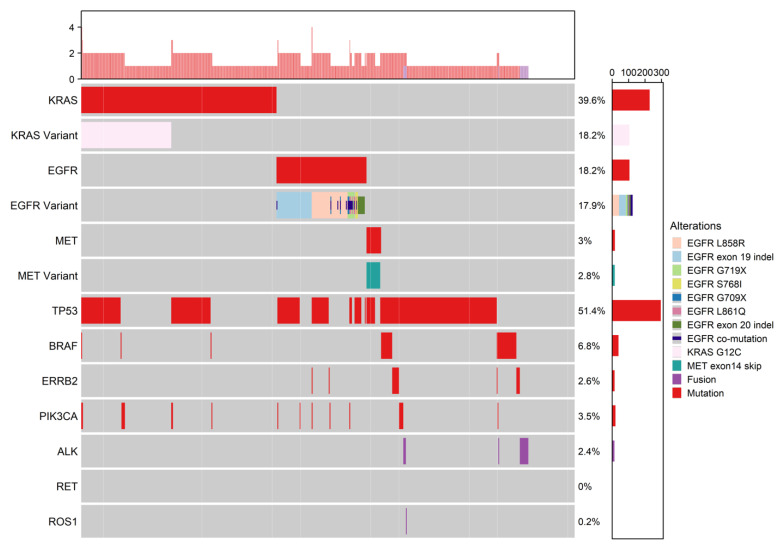
Oncoprint of the late-stage NSCLC cohort genomic alterations. This oncoprint depicts genetic alterations identified by the DNA-based hybrid-capture multiplex NGS assay (“oncopanel”) from the Cancer Genetics & Genomic Laboratory (CGL) at BC Cancer Vancouver Centre. ALK, RET, and ROS1 were detected with immunohistochemistry in this cohort. On the right, the alteration frequency per gene is reported, with the corresponding gene labelled on the left side. The barplots beside the gene labels report the alteration counts. The topmost bar represents the mutation and fusion count per sample.

Targeted sequencing of 59 early stage FFPE lung patient samples identified the most common mutations as EGFR (30.5%) common and uncommon variants, KRAS (28.8%) variants including KRAS G12C (13.6%), MET exon 14 skipping (5.1%), ERBB2 (3.4%), and gene fusions in ALK (1.7%), RET (3.4%), and ROS1 (1.7%) (Figure 1. early stage oncoprint, Table 3). In the 576 sample late-stage lung cohort, the most common mutations identified were also EGFR (18.2%) and KRAS (39.6%), including KRAS G12C (18.2%), MET exon 14 skipping (2.8%), ERBB2 (2.6%), ALK (2.4%), and ROS1 (0.2%) (Figure 2. late stage oncoprint, Table 3). Both cohorts showed similar frequencies of TP53 mutations with 45.8% in the early-stage cohort and 51.4% in the late-stage cohort. Mutations in PIK3CA (10.2% early stage, 3.5% late-stage) and BRAF (1.7% early stage, 6.8% late-stage) were also identified in both the early and late-stage cohorts at varying frequencies.

Unadjusted analysis revealed a significantly higher frequency of any EGFR mutations in the early-stage group compared to the late-stage group (30.5% versus 18.2%; *p* = 0.023) (Table 3). However, after matching on the propensity score for sex and smoking status, no significant difference in the EGFR mutation frequency was observed (32.1% versus 28.3%; *p* = 0.65) (Table 4). Unadjusted analysis also revealed a significantly higher frequency of uncommon EGFR mutations (tyro-sine kinase inhibitor (TKI) sensitizing mutations such as EGFR G719X, S768I, and L861Q) in the early-stage group compared to the late-stage group (8.5% versus 3.1%; *p* = 0.05) (Table 3). With propensity matching, no statistically significant difference remained for uncommon TKI-sensitive EGFR variants (EGFR 9.4% versus 4.4%; *p* = 0.17) (Table 4). The late-stage cohort harbored a higher frequency of KRAS variants (28.8% early stage vs. 39.6% late-stage); however, this was not statistically significant. There were also no statistical differences between the cohorts when comparing the frequency of gene mutations found in MET, ERBB2, TP53, ALK, RET, ROS1, PIK3CA, and BRAF.

A regression analysis was performed to summarize of the odds of mutations and fusion molecular alteration profiles comparing early to late-stage NSCLC for all patients and propensity-matched patients (Table 5). This analysis revealed similar odds of tumors with clinically actionable NSCLC molecular profiles between groups, notably for any EGFR mutation with adjustment for age, sex, and smoking status (*p* = 0.58). In contrast, odds of uncommon EGFR TKI-sensitive variants were increased in the early-stage group (*p* = 0.025).

## 4. Discussion

Multiple genetic alterations have been identified that impact the selection of systemic therapy to improve survival for patients with recurrent or late-stage NSCLC. Testing of tumor tissue for these alterations is important to identify potentially efficacious targeted therapy for patients, as well as to inform treatment plan thereby avoiding systemic therapeutic options unlikely to provide clinical benefit. Even early-stage lung cancers amenable to surgical resection have a high risk of recurrence, with 5-year survival ranging from 36% to 82% depending on NSCLC stage at presentation [4]. In the event of recurrent NSCLC, adequate tissue for molecular profiling diagnosis may be difficult with small needle biopsies or simply not accessible for biopsy due to anatomic location. This is a major limitation to obtaining important molecular testing information to guide treatment options, as these small samples may provide insufficient substrate for histologic, biomarker, and molecular testing. The very process of the need to re-acquire and await molecular testing results in the setting of disease relapse introduces important delays to timely delivery of systemic therapy.

While some centers are fortunate to have reflexive molecular testing workflows established postoperatively, many institutions still do not conduct reflexive molecular testing that includes an expansive cancer gene panel for resected early-stage NSCLC. Further to this, at institutions where molecular testing is conducted for resected early-stage NSCLC, it is often limited to common sensitizing EGFR variants. However, as determined in this study, uncommon TKI sensitizing EGFR variants such as EGFR G719X, S7681, and L861Q mutations were detected in the early-stage cohort, despite the small group size [21,22,23]. Systematic testing of large samples of FFPE tumor tissue from initial surgical resection for clinically actionable molecular alterations with an expanded targeted cancer gene panel-based approach for NGS is the ideal option to inform therapeutic options that will improve survival in the event of recurrence and avoid needless delay in systemic treatment selection. This is assuming that the frequency of such targetable alterations is similar in those with early and late-stage NSCLC. In this study, we provide the first report comparing the frequency of molecular alterations in early and late NSCLC in propensity-matched cohorts.

### 4.1. Molecular Alteration Frequency Profiles—Early versus Late-Stage NSCLC

The mutations most frequently reported in NSCLC occur in the KRAS and EGFR genes, which are typically mutually exclusive [14]. This was also observed in our matched early and late-stage cohorts, consistent with previous reports. Of interest, our late-stage cohort was noted to have an unadjusted comparatively high KRAS mutation frequency (for all variants) compared to the early-stage cohort. The presence of a KRAS mutation is a known prognostic marker of poor survival compared to patients whose tumors do not express KRAS [15]. The observed frequency of KRAS mutations in the late-stage sub-group is congruent with its status as a marker of aggressive tumor biology, portending poor clinical prognosis. We observed similar frequency of KRAS G12C in both the early and late cohorts. This is particularly relevant as novel systemic therapeutics are under development and USFDA approved to clinically target KRAS G12C [11,16,17].

EGFR mutations in lung adenocarcinoma are known to occur in 10–50% of NSCLC depending on the population, tending to occur more often in females, Asians, and never smokers [14]. These mutations typically cluster around exons 18, 19, 20, and 21. We observed a higher frequency of EGFR in the early-stage cohort compared to the later stage in the unadjusted analysis; however, this was not significantly different in pair-matched multivariable analysis. The higher observed frequency of EGFR in the unadjusted early-stage cohort may be related to the higher frequency of never smokers in this group. Never smoking status has previously been reported to be associated with EGFR expression [14,15]. Likewise, current or former smoker status has been reported to be associated with KRAS mutations in NSCLC.14 We did observe a higher frequency of current or former smokers in the late-stage cohort (64.4% early vs. 79.4% late).

All other frequencies were observed to be similar between the early and late-stage sub-groups. This included similar frequencies of the most common clinically actionable EGFR mutation variants (L858R and exon 19 deletion). These mutations confer sensitivity to targeted therapies with tyrosine kinase inhibitors (TKI), such as Osimertinib [24,25]. Interestingly, frequent EGFR insertion mutations in exon 20 were identified in the cohort. This is a clinically relevant finding, as such mutations may confer resistance to first line TKI systemic therapy [26]. However, the USFDA has recently approved the use of Mobocertinib as an irreversible TKI specifically for activating EGFR exon 20 insertion mutations, as well as the monoclonal antibody Amivantamab [27,28,29].

Genetic alterations in ALK occur in frequencies 2–7% of NSCLC patients, primarily chromosomal inversions or translocations that commonly result in the ALK-EML4 fusion [6,7,14]. ALK gene alterations noted in our report for both the early and late-stage sub-groups occurred at frequencies similar to that reported in the literature. Chromosomal rearrangements in the ROS1 proto-oncogene are reported to occur in approximately 1–2% of NSCLC patients, a frequency similarly observed in our early-stage cohort [14,30,31]. MET exon 14 “skipping” mutations occur in approximately 1–5% of NSCLC [14,32]. Similarly, the findings in our study are concordant with the frequency in the literature for both our early and late-stage sub-groups.

Such individuals with alterations in MET, ALK, and ROS1 may be eligible for treatment with targeted therapy [15,33].

RET rearrangements occur in 1–2% of NSCLC, portending sensitivity to a number of targeted inhibitors [34,35,36]. In the early stage cohort, we observed two patient samples both harboring KIF5B-RET fusions; however, we did not observe any RET-rearranged NSCLC in the late-stage cohort. This finding is highly clinically relevant, as previous reports show RET to be associated with potentially aggressive disease biology [33,34,35,36].

### 4.2. Study Limitations

Our findings are not without limitation, including the retrospective nature of the analysis which inherently introduced information bias despite the prospective nature of database variable procurement. The retrospective design also limits our assessment of tumor clonal heterogeneity both in space and time in that we are unable to map clonal evolution [37]. Furthermore, although we observed interesting molecular alteration profiles in our cohort, the small sample size of the early-stage sub-group in particular limits study power. A larger sample size is necessary to inform on the clinical relevance of the uncommon EGFR variants detected in our early-stage group. Additionally, this work represents the experience of a single large tertiary level regional thoracic surgical center of excellence. As such, the generalizability of these findings to the populations served by other treatment centers remains unknown.

### 4.3. Conclusions

Unadjusted analysis revealed higher frequency of common and uncommon sensitizing EGFR mutations in the early-stage NSCLC group. However, the propensity-matched analysis controlling for sex and smoking status demonstrated a similar frequency and odds of clinically actionable EGFR molecular alterations in early and late-stage NSCLC. This was in addition to the identification of many additional potential therapeutic targets, for example, KRAS G12C, MET exon 14 skipping, and gene fusion events in ALK, RET, and ROS1. Given the high risk of disease relapse even in those presenting with early-stage disease undergoing surgical resection, and similar clinically actionable mutation profiles compared to late-stage NSCLC, strong consideration should be given to reflexive panel-based targeted molecular profiling of FFPE tissue. With the adaptation of this molecular tumor tissue testing approach, more patients will derive clinical benefit from timely targeted systemic therapy delivery in case of subsequent relapse with the predetermined molecular profile of their tumor.

## Figures and Tables

**Table 1:** Baseline characteristics by stage of NSCLC among all patients.

Demographics/Diagnosis	All *n* = 635	Early Stage *n* = 59	Late Stage *n* = 576	*p* Value	Standardized Mean Difference (SMD)
Age (years): mean ± sd	70.9 ± 10.2	68.0 ± 10.3	71.2 ± 10.2	0.020	−31.2
Sex (female): n (%)	276 (43.5)	22 (37.3)	254 (44.1)	0.32	−11.4
Smoking status: n (%)				0.027	20.4
Never smoker	140 (22.0)	21 (35.6)	119 (20.7)		
Former smoker	385 (60.6)	31 (52.5)	354 (61.5)		
Current smoker	110 (17.3)	7 (11.9)	103 (17.9)		
Histology: n (%)				<0.001	72.4
Adenocarcinoma	527 (83.0)	49 (83.1)	478 (83.0)		
Squamous cell carcinoma	19 (3.0)	9 (15.3)	10 (1.7)		
Other lung carcinoma	89 (14.0)	1 (1.7)	88 (15.3)		

**Table 2:** Baseline characteristics by stage of NSCLC among matched patients.

Demographics/Diagnosis	All *n* = 212	Early Stage *n* = 53	Late Stage *n* = 159	*p* Value	Standardized Mean Difference (SMD)
Age (years): mean ± sd	68.5 ± 11.2	68.5 ± 10.2	68.5 ± 11.5	0.99	0.0
Sex (female): n (%)	143 (67.5)	34 (64.2)	109 (68.6)	0.43	−7.6
Smoking status: n (%)				0.83	9.8
Never smoker	83 (39.2)	21 (39.6)	62 (39.0)		
Former smoker	108 (50.9)	26 (49.1)	82 (51.6)		
Current smoker	21 (9.9)	6 (11.3)	15 (9.4)		
Histology: n (%)				0.99	3.1
Adenocarcinoma	197 (92.9)	49 (92.5)	148 (93.1)		
Squamous cell carcinoma	11 (5.2)	3 (5.7)	8 (5.0)		
Other lung carcinoma	4 (1.9)	1 (1.9)	3 (1.9)		

**Table 3:** Summary of outcomes among all patients.

Outcome Variable	All *n* = 635	Early Stage *n* = 59	Late Stage *n* = 576	*p* Value
Any alteration mutation or fusion	594 (93.5)	55 (93.2)	539 (93.6)	0.92
Potential therapeutic target	274 (43.1)	29 (49.2)	245 (42.5)	0.33
EGFR mutation present	123 (19.4)	18 (30.5)	105 (18.2)	0.023
EGFRm common sensitizing present	91 (14.3)	8 (13.6)	83 (14.4)	0.86
EGFR exon 19 deletion	45 (7.1)	4 (6.8)	41 (7.1)	0.92
EGFR L858R	46 (7.2)	4 (6.8)	42 (7.3)	0.89
EGFRm uncommon sensitizing present **	23 (3.6)	5 (8.5)	18 (3.1)	0.05
EGFR G709X *	5 (0.8)	1 (1.7)	4 (0.7)	0.39
EGFR G719X *	11 (1.7)	3 (5.1)	8 (1.4)	0.07
EGFR S768I *	4 (0.6)	1 (1.7)	3 (0.5)	0.99
EGFR L861Q/R *	6 (0.9)	0 (0.0)	6 (1.0)	0.32
EGFR co-mutation	15 (2.4)	2 (3.4)	13 (2.3)	0.64
EGFRm uncommon non-sensitizing present *	13 (2.0)	4 (6.8)	9 (1.6)	0.025
EGFR exon 20 insertion *	12 (1.9)	4 (6.8)	8 (1.4)	0.019
KRAS any mutation	245 (38.6)	17 (28.8)	228 (39.6)	0.11
KRAS G12C	113 (17.8)	8 (13.6)	105 (18.2)	0.37
Met present	20 (3.1)	3 (5.1)	17 (3.0)	0.42
MET exon14 skip	19 (3.0)	3 (5.1)	16 (2.8)	0.41
TP53 mutation	323 (50.9)	27 (45.8)	296 (51.4)	0.41
BRAF mutation *	40 (6.3)	1 (1.7)	39 (6.8)	0.16
ERRB2 mutation	17 (2.7)	2 (3.4)	15 (2.6)	0.67
PIK3CA mutation	26 (4.1)	6 (10.2)	20 (3.5)	0.026
FUSION present	19 (3.0)	4 (6.8)	15 (2.6)	0.09
ALK fusion *	15 (2.4)	1 (1.7)	14 (2.4)	0.99
RET fusion *	2 (0.3)	2 (3.4)	0 (0.0)	0.009
ROS1 fusion *	2 (0.3)	1 (1.7)	1 (0.2)	0.18

* Not to be compared further due to lack of events in the groups. ** Uncommon missense change in exon 21 of EGFR (also known as EGFR L861Q); uncommon EGFR G719X, S768I, and L861Q mutations. Values shown as n (%).

**Table 4:** Summary of outcomes among matched patients.

Alteration	All Matched *n* = 212	Early Stage *n* = 53	Late Stage *n* = 159	*p* Value
Any alteration mutation or fusion	199 (93.9)	50 (94.3)	149 (93.7)	0.87
Potential therapeutic target	106 (50.0)	29 (54.7)	77 (48.4)	0.44
EGFR mutation present	63 (29.7)	17 (32.1)	46 (28.9)	0.65
EGFRm common sensitizing present	44 (20.8)	8 (15.1)	36 (22.6)	0.23
EGFR exon 19 deletion	26 (12.3)	4 (7.5)	22 (13.8)	0.24
EGFR L858R	18 (8.5)	4 (7.5)	14 (8.8)	0.78
EGFRm uncommon sensitizing present **	12 (5.7)	5 (9.4)	7 (4.4)	0.17
EGFR G709X *	1 (0.5)	1 (1.9)	0 (0.0)	-
EGFR G719X *	6 (2.8)	3 (5.7)	3 (1.9)	-
EGFR S768I *	1 (0.5)	1 (1.9)	0 (0.0)	-
EGFR L861Q *	3 (1.4)	0 (0.0)	3 (1.9)	-
EGFR co-mutation	6 (2.8)	2 (3.8)	4 (2.5)	0.64
EGFRm uncommon non-sensitizing present *	8 (3.8)	4 (7.5)	4 (2.5)	-
EGFR exon 20 insertion *	8 (3.8)	4 (7.5)	4 (2.5)	-
KRAS any mutation	60 (28.3)	17 (32.1)	43 (27)	0.46
KRAS G12C	25 (11.8)	8 (15.1)	17 (10.7)	0.40
Met present	8 (3.8)	3 (5.7)	5 (3.1)	0.40
MET exon14 skip	8 (3.8)	3 (5.7)	5 (3.1)	0.40
TP53 mutation	96 (45.3)	23 (43.4)	73 (45.9)	0.75
BRAF mutation *	16 (7.5)	1 (1.9)	15 (9.4)	-
ERRB2 mutation	7 (3.3)	2 (3.8)	5 (3.1)	0.81
PIK3CA mutation	9 (4.2)	3 (5.7)	6 (3.8)	0.55
FUSION present	13 (6.1)	4 (7.5)	9 (5.7)	0.62
ALK fusion *	9 (4.2)	1 (1.9)	8 (5)	-
RET fusion *	2 (0.9)	2 (3.8)	0 (0.0)	-
ROS1 fusion *	2 (0.9)	1 (1.9)	1 (0.6)	-

* Not compared due to lack of events in the groups. ** Uncommon missense change in exon 21 of EGFR (also known as EGFR L861Q); uncommon EGFR G719X, S768I, and L861Q mutations. Values shown as n (%).

**Table 5:** Comparison of outcomes between early and late-stage among all and matched patients.

	All Patients				Matched Patients	
	Univariate Regression Analysis		Multiple Regression Analysis		Univariate Regression Analysis	
Alteration	Odds Ratio (95% CI) Late vs. Early	*p* Value	Odds Ratio (95% CI) Late vs. Early	*p* Value	Odds Ratio (95% CI) Late vs. Early	*p* Value
Any alteration mutation or fusion	1.059 (0.364, 3.083)	0.92	0.993 (0.354, 2.784)	0.99	0.895 (0.238, 3.359)	0.87
Potential target	0.766 (0.448, 1.309)	0.33	0.790 (0.439, 1.423)	0.43	0.788 (0.430, 1.445)	0.44
EGFR mutation present	0.508 (0.281, 0.919)	0.025	0.656 (0.34, 1.263)	0.21	0.849 (0.420, 1.719)	0.65
EGFRm common sensitizing present	1.073 (0.492, 2.343)	0.86	1.571 (0.687, 3.590)	0.28	1.715 (0.711, 4.137)	0.23
EGFR exon 19 deletion	1.054 (0.364, 3.052)	0.92	1.489 (0.512, 4.329)	0.46	1.940 (0.643, 5.853)	0.24
EGFR L858R	1.081 (0.374, 3.129)	0.89	1.282 (0.454, 3.621)	0.64	1.180 (0.373, 3.731)	0.78
EGFRm uncommon sensitizing present	0.348 (0.124, 0.975)	0.045	0.328 (0.121, 0.891)	0.029	0.411 (0.116, 1.460)	0.17
EGFR G709X *	-	-	-	-	-	-
EGFR G719X *	-	-	-	-	-	-
EGFR S768I *	-	-	-	-	-	-
EGFR L861Q *	-	-	-	-	-	-
EGFR co-mutation	0.658 (0.145, 2.989)	0.59	0.551 (0.138, 2.205)	0.40	0.667 (0.122, 3.640)	0.64
EGFRm uncommon non-sensitizing present **	-	-	-	-	-	-
EGFR exon 20 insertion *	-	-	-	-	-	-
KRAS any mutation	1.618 (0.899, 2.913)	0.11	1.071 (0.554, 2.070)	0.84	0.766 (0.376, 1.560)	0.46
KRAS G12C	1.421 (0.655, 3.083)	0.37	1.099 (0.501, 2.412)	0.81	0.685 (0.283, 1.659)	0.40
MET present	0.568 (0.161, 1.997)	0.38	0.505 (0.154, 1.657)	0.26	0.521 (0.113, 2.390)	0.40
MET exon14 skip	0.533 (0.151, 1.886)	0.33	0.475 (0.144, 1.569)	0.22	0.521 (0.113, 2.390)	0.40
TP53 mutation	1.252 (0.732, 2.144)	0.41	1.241 (0.697, 2.207)	0.46	1.110 (0.589, 2.091)	0.75
BRAF mutation *	-	-	-	-	-	-
ERRB2 mutation	0.762 (0.170, 3.414)	0.72	0.635 (0.161, 2.507)	0.52	0.795 (0.125, 5.077)	0.81
PIK3CA mutation	0.318 (0.122, 0.826)	0.019	0.430 (0.154, 1.201)	0.11	0.642 (0.150, 2.755)	0.55
FUSION present	0.368 (0.118, 1.146)	0.08	0.420 (0.138, 1.283)	0.13	0.737 (0.219, 2.485)	0.62
ALK fusion *	-	-	-	-	-	-
RET fusion *	-	-	-	-	-	-
ROS1 fusion *	-	-	-	-	-	-

* Not compared due to lack of events in the groups. ** Uncommon missense change in exon 21 of EGFR (also known as EGFR L861Q); uncommon EGFR G719X, S768I, and L861Q mutations.

## Data Availability

The data presented in this study are available on request from the corresponding author.

## Abbreviations

AJCC	American Joint Commission on Cancer
ALK	Anaplastic lymphoma kinase
EGFR	Epidermal growth factor receptor
FDG-PET	Fluorodeoxyglucose positron emission tomography
FFPE	Formalin fixed paraffin embedded
MET	Met proto-oncogene
NSCLC	Non-small cell lung cancer
RET	Ret proto-oncogene
ROS1	c-ROS proto- oncogene 1
SCLC	Small cell lung cancer (SCLC)
TKI	Tyrosine kinase inhibitor (TKI)
VCC	Vancouver Cancer Centre
VGH	Vancouver General Hospital
USFDA	United States Food and Drug Administration
VAF	Variant allele frequency
WHO	World Health Organization
